# MELD-Sarcopenia is Better than ALBI and MELD Score in Patients with Hepatocellular Carcinoma Awaiting Liver Transplantation

**DOI:** 10.31557/APJCP.2021.22.7.2005

**Published:** 2021-07

**Authors:** Ayman Alsebaey, Aliaa Aliaa, Hanaa Said Rashed, Maha Mohammad Elsabaawy, Amr Ragab, Rasha Abdelhafiz Aly, Hanaa Badran

**Affiliations:** 1 *Department of Hepatology and Gastroenterology, National Liver Institute, Menoufia University, Egypt. *; 2 *Department of Anesthesia, National Liver Institute, Menoufia University, Shebeen El-Koom, Egypt. *; 3 *Department of Diagnostic Medical Imaging and Interventional Radiology, National Liver Institute, Menoufia University, Shebeen El-Koom, Egypt. *

**Keywords:** MELD, MELD, sarcopenia, ALBI, sarcopenia, hepatocellular carcinoma

## Abstract

**Background:**

The albumin bilirubin (ALBI) score and model of end stage liver disease (MELD) are prognostic in patients with hepatocellular carcinoma (HCC). Aim was to compare MELD-sarcopenia to MELD and ALBI scores in patients with HCC awaiting liver transplantation.

**Methods:**

patients with HCC (n=262) were included and followed up for 12 months. Baseline MELD, ALBI and MELD-sarcopenia models were calculated.

**Results:**

The average age was 59.61 ±8.09 years. Most patients were males (69.5%), CTP class A (55.7%) and BCLC stage B (54.2%). Hepatitis C virus was the main cause of liver cirrhosis in most patients (88.9%). The average MELD, MELD-sarcopenia and median ALBI score were 10.65 ±2.54, 15.11 ±6.22 and -2.12 (0.74) respectively. Sarcopenia patients had higher MELD, ALBI and MELD-sarcopenia values. Patients with sarcopenia had lower survival (10.09 months) than those without (11.72 months). The ALBI, MELD and MELD-sarcopenia were associated with mortality. ALBI had AUROC of 0.717 (95% CI: 0.659 - 0.771), MELD had AUROC of 0.656 (95% CI: 0.595 - 0.713) and MELD-sarcopenia had AUROC of 0.798 (95% CI: 0.744 - 0.845). The ALBI and MELD scores had comparable AUROC (p=0.081). The MELD-sarcopenia had superior AUROC than MELD (p=0.001) and ALBI (p=0.05).

**Conclusion:**

MELD-sarcopenia is better prognostic model than the ALBI and MELD scores in HCC patients awaiting liver transplantation.

## Introduction

Hepatocellular carcinoma (HCC) is an ominous complication of chronic liver disease particularly cirrhosis (Forner et al., 2018). HCC is the 5th malignancy in men and the 8th in ladies. HCC is associated with decreased survival (Elshaarawy et al., 2019). Ahmad et al., (2020) followed up 83 patients, 50-70 years old, who underwent trans-arterial chemoembolization for 2 years to assess the survival. Most patients had HCV related cirrhosis and were Child-Pugh (CTP) class A. the baseline focal lesion size was 5-10cm in 45% of patients and mostly bilobar. Stable disease was detected in 27 patients, however, 18 and 21 patients had progression in the embolized lesions, and progression with new lesion formation, respectively. The 1- and 2-year survival was 80% and 56.6%, respectively.

The Barcelona clinic liver cancer (BCLC) model is based on various parameters as the Child-Pugh score, performance status, focal lesion size, number, metastasis, vascular invasion, and portal hypertension. BCLC classifies patients into five stages (0, A, B, C, D) and aids the decision of treatment strategy (Liver, 2018).

Sarcopenia is the loss of the muscle mass. It is a common complication of liver cirrhosis. Sarcopenia can be diagnosed by hand grip strength, frailty, dual-energy X-ray absorptiometry, appendicular lean mass, computerized tomography (CT) and magnetic resonance imaging. Sarcopenia is commonly associated with the development of hepatic encephalopathy, refractory ascites and the pre-transplant mortality (Sinclair et al., 2016; Rendina et al., 2019). The albumin bilirubin (ALBI) score and model of end stage liver disease (MELD) are based on routine daily investigations. They are independent predictors of the mortality in patients with HCC (Elshaarawy et al., 2019).

We aimed to compare the MELD-sarcopenia model to MELD and ALBI scores as a predictor of the survival in HCC patients awaiting liver transplantation.

## Materials and Methods

*Patients and Methods*

Our study was conducted in National Liver Institute hospitals, Menoufia University, Egypt. Our study was approved by the institutional review board. All patients signed informed written consent before enrollment.

Two hundred sixty-two patients with HCC were included (2017-2020). They were recruited from the liver transplantation clinic awaiting the availability of living donor and they were co-managed with the HCC multidisciplinary outpatient clinic team. 

HCC was diagnosed in accord to the characteristic triphasic CT criteria namely focal lesion arterial enhancement and portal/venous washout (Liver. and Cancer, 2012). All the patients underwent detailed history taking and complete physical examination. 

The following investigations were done; liver function tests, INR, CBC, serum creatinine and alfa fetoprotein (AFP). Patients were staged in accord to the BCLC staging system. The patients underwent different local or systemic treatment according to the multidisciplinary HCC team decision. The patients were followed up for one year.

Sarcopenia was assessed radiologically by studying the musculature at the level of third lumbar vertebra. 

Carey et al., (2017) proposed a skeletal muscle index ≤50 cm^2^/m^2^ for men and ≤39 cm^2^/m^2^ for women as the definition of sarcopenia in patients with end-stage liver disease awaiting liver transplantation.

*Calculations*

Model for end-stage liver disease (MELD) (Abdelsameea et al., 2020)=3:78 ln [serum total bilirubin (mg/dL)] + 11:2 ln [INR] + 9:57 ln [serum creatinine (mg/dL)] + 6:43.

MELD-sarcopenia (Montano-Loza et al., 2015; van Vugt et al., 2018) =MELD + (10.35 * Sarcopenia)

Albumin-bilirubin (ALBI) score (Johnson et al., 2015)= −0.085 × (albumin g/L) + 0.66 × log (bilirubin μmol/L).

*Statistical Analysis*

Mean ±standard deviation or median ± interquartile range was used for continuous variables and percentages were used for nominal variables. Comparisons between two groups were performed using the student’s t-test for parametric analysis, Mann-Whitney test for nonparametric analysis and chi-squared or Fisher exact test for analyzing categorical data. Survival curves were plotted using Kaplan-Meier analysis and compared by log-rank test. The effect of examined independent variables in the survival was estimated by a Cox proportional-hazards regression. The Receiver Operating Characteristic (ROC) curve was used to quantify the prognostic value of presented scores and differences between them was calculated by the area under the ROC (AUROC) according to deLong method. Statistically significant was considered a two-tailed p-value less than 0.05. Data was statistically analyzed using IBM® SPSS® Statistics® version 21 for Windows.

## Results

In our study, as shown in Table 1, the mean age was 59.61 ±8.09 years with median AFP of 41 (101.7) ng/ml. Most patients were males (69.5%), CTP class A (55.7%) and BCLC stage B (54.2%). Hepatitis C virus was the main cause of liver cirrhosis in most patients (88.9%).

The mean MELD, MELD-sarcopenia and median ALBI score were 10.65 ±2.54, 15.11 ±6.22 and -2.12 (0.74) respectively.

As demonstrated in Table 2, patients with sarcopenia had statistically significantly (p=0.001) higher values of MELD (11.29 ±2.43 vs 10.15 ±2.52), ALBI [-2 (0.68) vs -2.23 (0.75)] and MELD-sarcopenia (21.64 ±2.43 vs 10.15 ±2.52) than those without sarcopenia.

**Figure 1:**
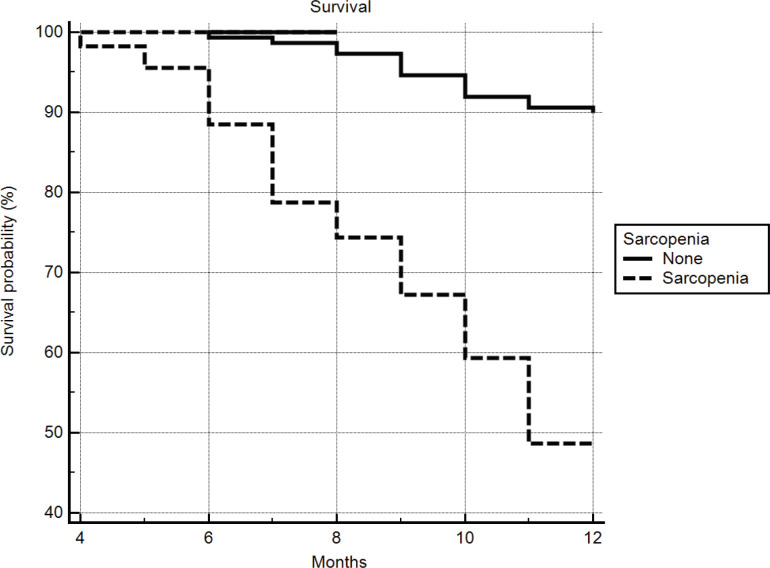
Kaplan-Meier Survival Analysis

Patients with sarcopenia had lower survival (p=0.001) than those without; 10.09 months (95% CI: 9.65 - 10.53) and 11.72 months (95% CI: 11.57 - 11.88) respectively (Figure 1).

Instead of BCLC A/B, the ALBI, MELD and MELD-sarcopenia were statistically (p=<0.005) associated with the mortality (Table 3).

**Figure 2:**
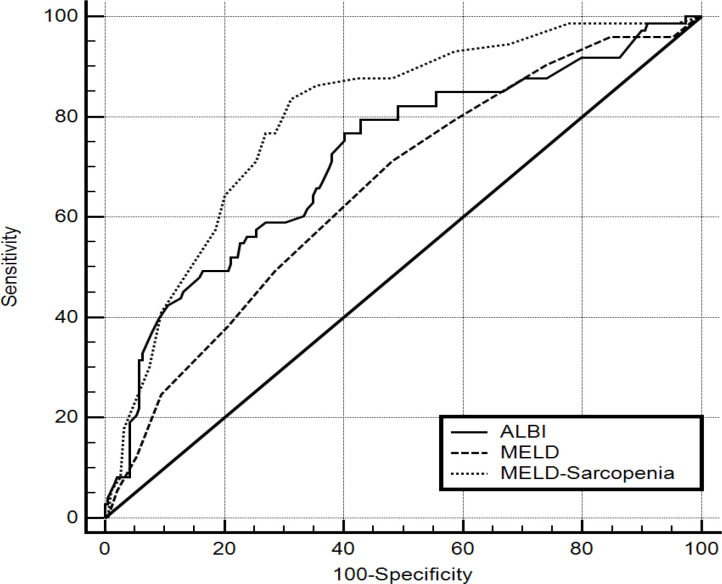
ROC Curve Analysis of the Prognostic Models in Patients with and without Sarcopenia

The ROC curve (Figure 2) showed that ALBI had AUROC of 0.717 (95% CI: 0.659 - 0.771), MELD had AUROC of 0.656 (95% CI: 0.595 - 0.713) and MELD-sarcopenia had AUROC of 0.798 (95% CI: 0.744 - 0.845).

The ALBI and MELD score had comparable AUROC (p=0.081). The MELD-sarcopenia had superior AUROC than MELD (p=0.001) and ALBI (p=0.05).

**Table 1:** Baseline Data

Age years		59.61 ±8.09
MELD		10.65 ±2.54
ALBI		-2.12 (0.74)
MELD-sarcopenia		15.11 ±6.22
AFP ng/ml		41 (101.7)
BCLC	BCLC A	74 (28.2%)
	BCLC B	142 (54.2%)
	BCLC C	46 (17.6%)
Sarcopenia		
	None	149 (56.9%)
	Sarcopenia	113 (43.1%)
Etiology		
	Viral negative	6 (2.3%)
	HBV	17 (6.5%)
	HCV	233 (88.9%)
	NASH	5 (1.9%)
	Others	1 (0.4%)
Sex	Female	80 (30.5%)
	Male	182 (69.5%)
CTP class	CTP A	146 (55.7%)
	CTP B	116 (44.3%)

Data are expressed as mean ±standard deviation, median (interquartile range) or number (column percentage)

**Table 2:** Comparison of the Prognostic Models in Patients with and witohout Sarcopenia

	Sarcopenia		P
	None N=149	Sarcopenia N=113	
MELD	10.15 ±2.52	11.29 ±2.43	0.001
ALBI	-2.23 (0.75)	-2 (0.68)	0.001
MELD-sarcopenia	10.15 ±2.52	21.64 ±2.43	0.001
BCLC A/B/C%	37.6/53/9.4	15.9/55.8/28.3	0.001

Data are expressed as mean ±standard deviation, median (interquartile range)

**Table 3:** The Cox Proportional-Hazards Regression Analysis of the Mortality

	B	P	Hazard Ratio	95% CI
MELD	0.17	0.0002	1.19	1.09 - 1.30
ALBI	1.56	<0.0001	4.77	2.68 - 8.49
MELD sarcopenia	1.16	<0.0001	1.17	1.12 - 1.22
BCLC B	1.56	0.0032	4.76	1.68 - 13.42
BCLC C	3.18	<0.0001	23.98	8.51 - 67.57

## Discussion

The CTP score was the first score to assess the prognosis of liver cirrhosis patients. The CTP included bilirubin, albumin, INR, ascites, and encephalopathy. The cutoff values were empirically selected and there are two components (ascites and encephalopathy) that were subjective. It did not include important parameters as the nutritional status, creatinine, and sodium level. Later, the MELD score that depends on serum bilirubin, INR and creatinine was adopted to assess mortality in transjugular intrahepatic portosystemic shunt “TIPS” patients and liver transplantation patients. But it neglected also the nutritional status (Durand and Valla, 2008).

The ALBI score was coined by Johnson et al, (Johnson et al., 2015) who assessed the liver functions in HCC patients and their correlation to the survival. Later, it was assessed in the diagnosis and the management of HCC patients as with radiofrequency or radioembolization (Hiraoka et al., 2017a; Hiraoka et al., 2017b; Kao et al., 2017; Antkowiak et al., 2019; Elshaarawy et al., 2019).

Montano-Loza et al (Montano-Loza et al., 2015) studied 669 patients with liver cirrhosis who are undergoing assessment for liver transplantation. About 45% of the patients were sarcopenic by CT criteria. Patients with sarcopenia had lower survival than those without. They developed a new score that was called the MELD-sarcopenia score. By comparison of the c-statistics; MELD-sarcopenia was superior to MELD in the prediction of the mortality.

In another recent study (van Vugt et al., 2018) that included 585 patients (33% had HCC), 43.4% had sarcopenia by the characteristic CT finding. The 3 months survival was lower in patients with sarcopenia. They found that the c-index of the MELD was superior to MELD-sarcopenia (0.839 vs 0.820).

In our study patients with sarcopenia had worse values of ALBI, MELD and MELD-sarcopenia score. Both Montano-Loza et al (Montano-Loza et al., 2015) and van Vugt et al., (2018) found higher MELD score in the sarcopenic patients.

Patients with baseline sarcopenia had lower survival compared to those without sarcopenia in accord with Montano-Leza et al., (2015) and van Vugt et al., (2018).

On comparison of AUROC the 3 studied score, MELD-sarcopenia was better than ALBI (0.798 vs 0.717) and MELD score (0.798 vs 0.656). Also, ALBI was better than the MELD score. Our finding regarding MELD-sarcopenia was in accord with Montano-Loza et al., (2015) and in disagreement with van Vugt et al., (2018). We do not have explanation for the disagreement with van Vugt et al., (2018).

Points of strength: we conducted the study mainly in HCC patients. Also, we have also tested the ALBI score. The limitations of the current study are that it is single center experienced and the patients’ number is relatively small.

In conclusion, MELD-sarcopenia is superior to ALBI and MELD scores for assessing the survival in HCC patients waiting liver transplantation.

*Abbreviations*

AFP; alfa fetoprotein, ALBI; albumin bilirubin score, BCLC; Barcelona clinic liver cancer, CT; computerized tomography, CTP; Child-Pugh score, HCC; hepatocellular carcinoma, MELD; model of end stage liver disease

## Author Contribution Statement

Ayman Alsebaey, Aliaa Sabry, Hanaa Said Rashed, Amr Ragab and Rasha Abdelhafiz Aly did the data collection, conception of the design, analysis, and paper writing. Final revision was done by both Maha Mohammad Elsabaawy and Hanaa Badran, and it was approved by all authors.
